# Testing for Antibodies to Four Parasites in Residual Blood Specimens from Trachoma Surveys in Kiribati, 2015–2019

**DOI:** 10.4269/ajtmh.23-0565

**Published:** 2025-02-11

**Authors:** AdeSubomi O. Adeyemo, Raebwebwe Taoaba, Diana Martin, E. Brook Goodhew, Robert Butcher, Caleb Mpyet, Emma Harding-Esch, Anasaini Cama, Sarah Anne J. Guagliardo, Sarah Gwyn, Anthony W. Solomon, Kabiri Tuneti Itaaka, Ana Bakhtiari, Cristina Jimenez, Rabebe Tekeraoi

**Affiliations:** ^1^ Centers for Disease Control and Prevention, Atlanta, Georgia;; ^2^ Epidemic Intelligence Service, Centers for Disease Control and Prevention, Atlanta, Georgia;; ^3^ Ministry of Health and Medical Services, South Tarawa, Kiribati;; ^4^ London School of Hygiene & Tropical Medicine, London, United Kingdom;; ^5^ Sightsavers, Nigeria Country Office, Kaduna, Nigeria;; ^6^ The Fred Hollows Foundation, Melbourne, Australia;; ^7^ Global Neglected Tropical Diseases Programme, World Health Organization, Geneva, Switzerland;; ^8^ Task Force for Global Health, Decatur, Georgia;; ^9^ Sightsavers, Haywards Heath, United Kingdom

## Abstract

To assess the prevalence of several parasitic infections in Kiribati, dried blood spots collected during trachoma prevalence surveys in the two major population centers in 2015, 2016, and 2019 were tested using multiplex bead-based serologic assays to detect IgG antibodies against four pathogens of public health interest: *Toxoplasma gondii* (*T. gondii*), *Taenia solium* (*T. solium*), *Strongyloides stercoralis* (*S. stercoralis*), and *Toxocara canis* (*T. canis*). In Kiritimati Island, the seroprevalences of *T. solium* recombinant antigen for detection of cysticercosis antibodies (T24H) and recombinant antigen for detection of taeniasis antibodies (ES33) were ≤4% in both surveys, whereas in Tarawa, the T24H seroprevalence was 2% (2016) and 7% (2019) and the ES33 seroprevalence was ≤3% in both surveys. At both sites, the seropositivity of *S. stercoralis* recombinant antigen for detection of *Strongyloides* was 0–4%, and for *T. canis*, the C-type lectin-1 antigen was 0–1% in all surveys. For *T. gondii*, the surface antigen glycoprotein 2A antigen seroprevalences on Kiritimati Island were 41% (2015) and 36% (2019), and in Tarawa, they were 36% (2016) and 22% (2019), suggesting that *T. gondii* infections are common in Kiribati, whereas the other pathogens are not.

Multiplex bead-based serologic assays (MBAs) can assess for antibodies to multiple pathogens in a single specimen. Testing specimens collected during single-disease surveys using MBAs can cost-effectively generate seroprevalence estimates for neglected diseases that might otherwise not be assessed, for example, because of a lack of data on the burden of infection or a lack of resources. In this study, we took advantage of dried blood spot (DBS) specimens collected during routine trachoma prevalence surveys in the remote island nation of Kiribati to assess for antibodies against antigens derived from *Strongyloides stercoralis* (*S. stercoralis*), *Toxocara canis* (*T. canis*),* Taenia solium* (*T. solium*), and *Toxoplasma gondii* (*T. gondii*) using MBA. Few data on the presence of these pathogens in Kiribati existed before our evaluation; therefore, public health officials were interested in obtaining prevalence estimates.

Baseline trachoma surveys were conducted in the two main population centers in Kiribati—Kiritimati Island (2015) and Tarawa (2016)— during which DBS specimens from children aged 1–9 years were collected.^1^^,^^2^ These surveys were powered to detect a follicular prevalence of trachomatous inflammation of 10% in 1- to 9-year-olds with a precision of 3%.^3^ Based on these survey results, interventions were implemented in 2017 and 2018 that included the mass drug administration of azithromycin (or tetracycline eye ointment for those in whom azithromycin was contraindicated), as well as efforts to enhance facial cleanliness and encourage environmental improvement. Follow-up trachoma impact surveys were conducted at each site in 2019 using a two-stage cluster-sampled population-based design adjusted for finite population size that was powered to detect a follicular prevalence of trachomatous inflammation of 4% in 1- to 9-year-olds with a precision of ±2%.^4^ The islands are >3,000 km from each other; therefore, data from each evaluation unit (EU) were assessed separately. In the clean dataset, 382 and 658 Kiritimati children contributed data for 2015 and 2019, respectively, and 863 and 867 Tarawa children contributed data for 2016 and 2019, respectively.

Finger-prick blood samples were collected onto filter paper extensions calibrated to hold 10 µL of blood (TropBio Pty Ltd., Queensland, Australia). The papers were air-dried overnight, placed in sealed plastic bags containing desiccant, and stored at −20°C until they were shipped at ambient temperature. Samples from DBS extensions were tested with MBA using previously described methods.^5^ The assay tested for total IgG antibodies to recombinant antigen for detection of *Strongyloides* (NIE) from *S. stercoralis*, recombinant antigen for detection of *Toxocara* (CTL-1) from *T. canis*, recombinant antigen for detection of cysticercosis (T24H) and recombinant antigen for detection of taeniasis (ES33) from *T. solium* (associated with the presence of tissue cysts and adult stage tapeworm, respectively),^6^ and recombinant antigen for detection of acute *Toxoplasma* (SAG2A) from *T. gondii*. A Bio-Plex 200 instrument (Bio-Rad, Hercules, CA) was used to read plates using the Bio-Plex manager 6.0 software (Bio-Rad). The antibody levels of each antigen were reported as median fluorescence intensity, with background fluorescence subtracted. The median fluorescence intensity with background fluorescence subtracted data were converted to seropositive/seronegative outcomes using the following cutoffs: 1,515 (NIE), 348 (CTL-1), 61 (ES33), 261 (T24H), and 163 (SAG2A). For CTL-1, the cutoff was calculated as three standard deviations greater than the mean response of a panel of 86 sera from US nontravelers. For all other antigens, cutoffs were calculated with a receiver operator characteristic (ROC) analysis using panels of defined positive sera from confirmed cases for each pathogen (or the presence of cysts for T24H) and negative sera from US nontravelers. Based on these ROC panels, the sensitivity and specificity are provided in Table 1. Sensitivity and specificity values were not available for CTL-1 because of the lack of defined panels; however, based on a previously reported study using these antigens, the sensitivity to detect visceral larva migrans was 90% (95% CI 85–94%), the sensitivity to detect ocular larva migrans was 54% (95% CI 39–68%), and the specificity was 99% (95% CI 97–100%).^7^ We used R version 4.0.3 (R Foundation for Statistical Computing, Vienna, Austria) for the statistical analysis. The seroprevalences at the two time points (baseline and impact surveys) were assessed for the two EUs, Kiritimati Island and Tarawa. The seroprevalence among 1- to 9-year-olds was further assessed in 3-year age bands. A χ^2^ test was used to compare the seroprevalences over time.

**Table 1:** Sensitivity and specificity for NIE, ES33, T24H, and SAG2 antigens

Antigen	Sensitivity (%)	95% CI	Specificity (%)	95% CI
NIE	83.9	72.8–91.0	100	95.7–100.0
ES33	90.6	75.8–96.8	98.9	93.8–99.9
T24H	92.6	84.8–96.6	96.5	90.2–99.1
SAG2	100	67.6–100.0	100	67.6–100.0

ES33 = recombinant antigen for detection of taeniasis antibodies; NIE = recombinant antigen for detection of *Strongyloides* antibodies; SAG2 = recombinant antigen for detection of acute *Toxoplasma* antibodies; T24H = recombinant antigen for detection of cysticercosis antibodies.

*Toxoplasma gondii* was the only pathogen for which a substantial seroprevalence was identified, with more than one-third of 1- to 9-year-olds testing seropositive for antibodies to SAG2A at baseline in both EUs (Table 2). In Tarawa, there was a significant decrease in antibody seroprevalence to *T. gondii* SAG2A, from 36% in 2016 to 22% in 2019 (χ^2^ = 40.8; *P*-value <0.001). A slight but not significant decrease in *T. gondii* SAG2A seroprevalence was observed on Kiritimati Island (Table 2). There was, however, a significant decrease in *T. gondii* SAG2A seroprevalence among the youngest children in both EUs (Table 3). Kiribati has a high feral cat population, which may lead to high rates of *T. gondii* infection through the ingestion of untreated water or raw or undercooked food.^8^ Although previous information on acute toxoplasmosis in Kiribati is unavailable, among primary school children aged ≤9 years in the Pacific Island nation of the Marshall Islands, there was an acute/recurrent infection rate of 40.74% (*n* = 81).^9^ The study in the Marshall Islands noted a positive but not statistically significant association of *T. gondii* infection among primary school children residing in homes with cats.^9^ Children in the 1–3 years age range in Kiribati may have been the most likely to have lower exposure to *T. gondii*, and hence lower seroprevalence, because of efforts to control the feral cat population, improved practices to prevent the accidental ingestion of contaminated soil, and (possibly) interventions for trachoma because azithromycin has modest effects against *T. gondii*, and hygiene interventions may have limited transmission.^10^

**Table 2:** Seroprevalence for antibodies to parasitic diseases antigens in 1- to 9-year-olds at trachoma baseline (Kirimati 2015, Tarawa, 2016) and impact (2019) surveys

Parasite	Antigen	Kiritimati Island						Tarawa					
		Year	*n*	%	95% CI	χ^2^; df = 1	*P*-Value	Year	*n*	%	95% CI	χ^2^; df = 1	*P*-Value
*Toxoplama gondii*	SAG2A	2015	382	41	37–46	3.2	0.07	2016	863	36	33–39	40.8	<0.001*
		2019	658	36	32–39			2019	867	22	19–25		
*Taenia solium*	ES33	2015	382	2	1–3	0	1	2016	863	1	1–2	2.39	0.12
		2019	658	1	1–3			2019	867	3	2–4		
	T24H	2015	382	2	1–4	2.9	0.09	2016	863	2	1–3	26.1	<0.001*
		2019	658	4	3–6			2019	867	7	6–9		
*Strongyloides stercoralis*	NIE	2015	382	0	0–1	0.1	0.73	2016	863	4	3–5	2.5	0.11
		2019	658	0	0–1			2019	867	2	1–3		
*Toxocara canis*	CTL	2015	382	0	0–1	N/A	N/A	2016	863	1	0–2	0.3	0.61
		2019	658	0	0–1			2019	867	1	1–2		

CTL = recombinant antigen for detection of *Toxocara* antibodies; df = degrees of freedom; ES33 = recombinant antigen for detection of taeniasis antibodies; NIE = recombinant antigen for detection of *Strongyloides* antibodies; SAG2 = recombinant antigen for detection of acute *Toxoplasma* antibodies; T24H = recombinant antigen for detection of cysticercosis antibodies.

* Significantly different seroprevalence for antibodies between baseline and impact surveys.

**Table 3:** Seroprevalence for antibodies to *Toxoplasma gondii* SAG2A antigen for baseline (Kirimati 2015, Tarawa, 2016) and impact (2019) surveys among children aged 1–9 years, by age group

Age Group	Kiritimati Island						Tarawa					
	Year	*n*	%	95% CI	χ^2^; df = 1	*P*-Value	Year	*n*	%	95% CI	χ^2^; df = 1	*P*-Value
1–3 years	2015	137	28	21–36	15.44	<0.001*	2016	234	17	12–22	11.82	<0.001*
	2019	233	11	8–16			2019	368	7	5–10		
4–6 years	2015	119	43	34–52	0.63	0.43	2016	381	37	34–42	7.72	0.005*
	2019	248	38	32–44			2019	316	27	22–32		
7–9 years	2015	126	55	46–63	2.47	0.12	2016	248	52	46–58	3.78	0.05
	2019	177	64	57–71			2019	183	42	35–49		

df = degrees of freedom; SAG2 = recombinant antigen for detection of acute *Toxoplasma* antibodies.

* Significantly different seroprevalence for antibodies between baseline and impact surveys.

The seroprevalences of *T. solium* T24H on Kiritimati Island increased modestly between 2015 and 2019 (2% versus 4%; *P* = 0.09) and more substantially in Tarawa between 2016 and 2019 (2% versus 7%; χ^2^ = 26.1; *P* <0.001; Table 2). These prevalences fall at the lower end of those observed in sero-epidemiological studies using an ELISA or enzyme-linked immunoelectrotransfer blot.^11^ However, no serological thresholds define *T. solium* as a public health concern. Because the prevalence of symptomatic neurocysticercosis increases with age in endemic areas,^12^^,^^13^ this study may underestimate the overall seroprevalence in the population. We found low seroprevalences (1–3%) of *T. solium* ES33 for both EUs. Although both estimates fall within the specificity range of the assay, in 2019, Tarawa demonstrated a 3% seroprevalence of the infection marker ES33 and a 7% seroprevalence of the T24H antigen, which has been associated with the presence of cysts. Therefore, follow-up may be indicated in Tarawa, particularly among people with epilepsy. Seropositivity was low for antibodies against NIE from *S. stercoralis* (≤4%) and CTL-1 from *T. canis* (≤1%) at both sites in each survey (Table 2). We found little evidence suggesting the significant transmission of either infection in Kiribati. Again, noting the specificity range of the assays concerned, the antibody signals observed against *S. stercoralis* and *T. canis* antigens could be attributable to false positives.

There are some limitations to these analyses. The age range examined is limited because of the nature of trachoma surveys, and because these pathogens are also prevalent in adult populations, these data may underestimate the seroprevalence in the total population. Second, although most of these antigens are well-characterized, ES33 has limited validation data. The CTL-1 antigen has poor sensitivity for ocular larva migrans; thus, antibodies against this antigen may underestimate transmission.^7^ Additionally, the foci of transmission for these pathogens may have been missed, although the data are strengthened by two independent cross-sectional sampling frames, which increases the combined population coverage.

Despite these limitations, we were able to generate the first published estimates of seroprevalence of *T. gondii*, *T. solium*, *S. stercoralis*, and *T. canis* in Kiribati via secondary testing of specimens from what otherwise would have been single-disease surveys. We were unable to identify any previous studies that assessed the impact of trachoma control and elimination programs on *T. gondii* seroprevalence or disease. However, through this study, we demonstrated that almost one-third of children in Kiribati were exposed to *T. gondii* and that there were marginal but significant decreases in *T. gondii* seroprevalence between baseline and follow-up. These decreases are potentially due to the antibiotic and hygiene interventions implemented for trachoma elimination, although any attribution of causality could only be speculative. The potential application of MBA is broad; assays have been explored for the simultaneous surveillance of infectious diseases, including HIV, viral hepatitis, syphilis, and herpes.^14^ Multiplex assays have also been explored to simultaneously screen for vaccine-preventable diseases, including measles and rubella, to guide immunization activities, including identifying lapses in coverage in a population.^15^^,^^16^ Through this study, we were able to demonstrate the value of adding multiplex serological testing to routine programmatic activity in assessing diseases of public health interest. Future larger-scale studies involving children and adult populations in Kiribati could further support the effectiveness of utilizing MBA to detect multiple pathogens while providing a representative sample of parasitic infections in the country.
